# Paediatric cranial ultrasound: assessment of the preterm brain

**DOI:** 10.1186/s13244-025-02030-5

**Published:** 2025-07-22

**Authors:** Caoilfhionn Ní Leidhin, Michael Paddock, Paul M. Parizel, Richard R. Warne, Peter Shipman, Rahul Lakshmanan

**Affiliations:** 1 Department of Medical Imaging, Perth Children’s Hospital, Perth, Western Australia Australia; 2 Perth Radiological Clinic, Perth, Western Australia Australia; 3 SKG Radiology, Subiaco, Western Australia Australia; 4 School of Medicine, The University of Notre Dame Australia, Fremantle, Western Australia Australia; 5 School of Medicine & Population Health, University of Sheffield, Sheffield, United Kingdom; 6 Medical School, University of Western Australia, Perth, Western Australia Australia; 7 Department of Radiology, Royal Perth Hospital (RPH), Perth, Western Australia Australia; 8 Perron Institute, Faculty of Medicine, Centre of Neurological and Neuromuscular Disorders, University of Western Australia, Perth, Western Australia Australia

**Keywords:** Head, Brain, Ultrasonography, Infant, Pathology

## Abstract

**Abstract:**

Cranial ultrasound is an invaluable tool in assessing neonatal brain anatomy and pathology. It is accessible, relatively quick, inexpensive, safe, portable and generally well-tolerated. This pictorial review focuses on the use of cranial ultrasound in evaluating the premature brain. We illustrate the different grades of intraventricular haemorrhage, the most common sequela of prematurity, its evolution and potential complications, as well as periventricular leukomalacia. Anatomical variants and benign findings that mimic preterm brain injury are also discussed.

**Critical relevance statement:**

Cranial US is an invaluable tool for assessing neonatal brain anatomy and pathology and can be used in preterm infants to diagnose, monitor and assess for complications of intraventricular haemorrhage and periventricular leukomalacia.

**Key Points:**

- Cranial US (CUS) is an invaluable tool for assessing the neonatal brain and has many advantages over MRI.
- CUS can detect intraventricular haemorrhage and periventricular leukomalacia, the most important sequelae of prematurity.
- Knowledge of optimal CUS technique, normal anatomy, and variants/benign sonographic findings that mimic pathology is crucial to avoid misdiagnosis and unnecessary concern.

**Graphical Abstract:**

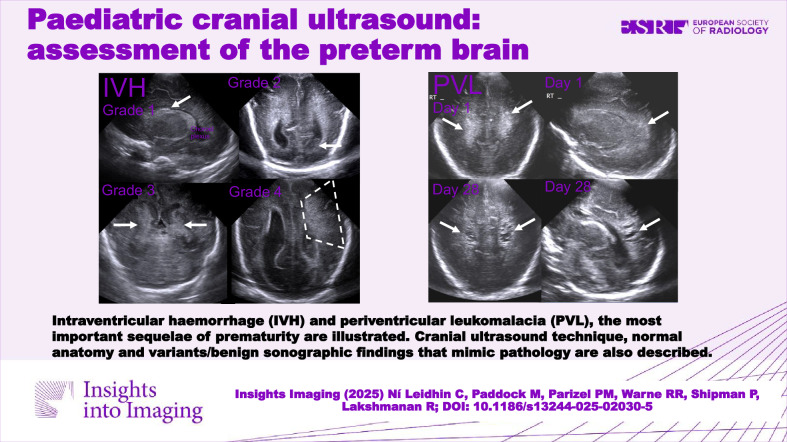

## Introduction

Cranial ultrasound (CUS) is a widely established tool for evaluating the neonatal brain. It is accessible, more affordable than magnetic resonance imaging (MRI), and can be performed at the cot-side in special care or neonatal intensive care units, which is important given the inherent risks associated with moving and transporting extremely premature neonates. Furthermore, it does not involve ionising radiation, is generally well-tolerated (even by critically ill and extremely premature babies) and does not require sedation or general anaesthesia. Due to its accessibility and ease of repetition, CUS is ideal for ongoing monitoring.

## Standard imaging protocol for the preterm brain

CUS is typically performed with a convex or curved linear medium-frequency (i.e., 3 to 10 mHz) transducer through the anterior fontanelle in both coronal and sagittal planes [1]. A higher-frequency linear transducer (i.e., 6 to 15 mHz) may also be used to assess superficial structures, such as the cerebral cortex, extra-axial spaces, and superior sagittal sinus [2, 3]. Transmastoid views are helpful in evaluating the posterior fossa [4].

**Fig. 1:**
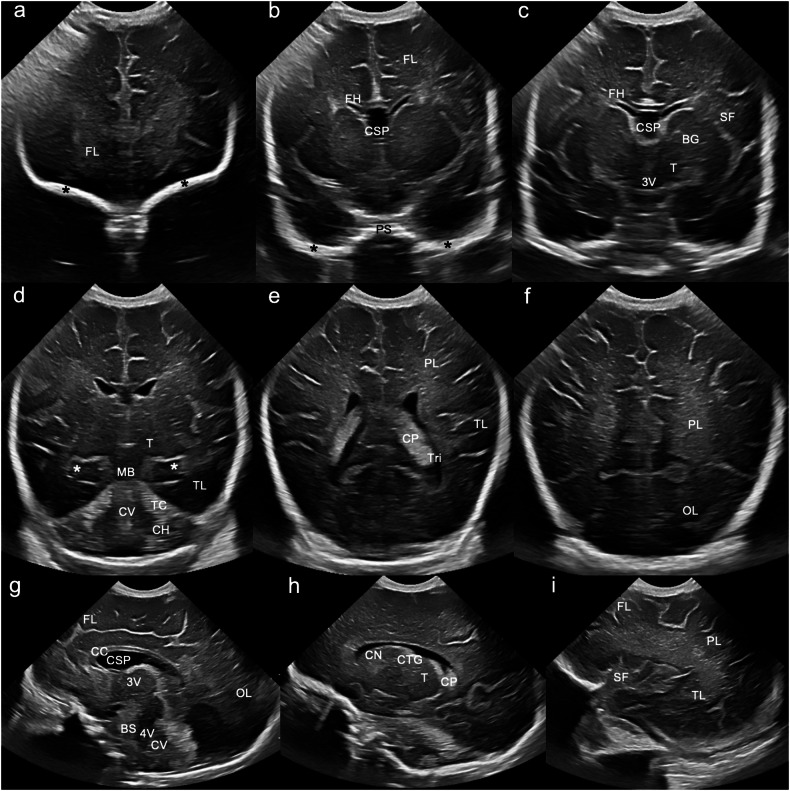
Standard sonographic views through the anterior fontanelle. Six coronal and five sagittal images are acquired: **a** The first coronal image depicts the frontal lobes (FL). The bony orbital roofs (*) in the far field act as a landmark. Note the hypoechoic grey matter peripherally and the hyperechoic subcortical and deep white matter centrally. **b** The second coronal image depicts the frontal lobes and frontal horns of the lateral ventricles (FH). Note the cavum septum pellucidum (CSP) interposed between the two frontal horns. The greater wings of sphenoid (*) and planum sphenoidale (PS) are visible in the far field. **c** The third coronal image is at the level of the third ventricle (3V) and includes the frontal horns of the lateral ventricles (FH), basal ganglia (BG)/thalami (T) and Sylvian fissures (SF). **d** The fourth coronal image is at the level of the cerebellum, and depicts the temporal lobes (TL), thalami (T), midbrain (MB), tentorium cerebelli (TC), cerebellar vermis (CV), and cerebellar hemispheres (CH). The far-field “C shape” of the parahippocampi and medial temporal lobes (*) serves as a landmark. **e** The fifth coronal image depicts the parietal (PL) and temporal lobes (TL) at the level of the lateral ventricular trigones (Tri). Note the echogenic choroid plexus (CP) within the lateral ventricular bodies. **f** The sixth and final coronal image depicts the parietal (PL) and occipital lobes (OL). **g** The first sagittal image depicts the midline structures—the corpus callosum (CC), cavum septum pellucidum (CSP), third (3V) and fourth ventricles (4V), brainstem, and cerebellar vermis (CV). Note the slightly increased echogenicity of the third and fourth ventricles compared to the CSP due to partial volume averaging. **h** The second sagittal image is the first parasagittal view, showing the “C-shaped” lateral ventricle, with the caudothalamic groove (CTG) separating the caudate nucleus (CN) anteriorly from the thalamus (T) posteriorly. Note the echogenic choroid plexus within the lateral ventricular body. **i** The third sagittal image is the second parasagittal image at the level of the Sylvian fissure (SF), depicting the frontal (FL), parietal (PL) and temporal (TL) lobes. **h** and **i** are repeated on the contralateral side to complete the sagittal imaging assessment

The standard CUS protocol includes six coronal/coronal oblique and five sagittal/sagittal oblique images (Fig. 1). For reliable interpretation, it is crucial that images are acquired symmetrically [5]. CUS is a dynamic, interactive examination, and saved still images should be considered representative. Cinematic video capture is a useful technique that allows dynamic evaluation of the brain parenchyma, improving diagnostic confidence and saving time [6], particularly when the person performing the examination may not be the one reporting it. While recently published multi-society recommendations suggest using Doppler routinely in preterm infants [7], judicious use of Doppler in this cohort is crucial, and pulse wave Doppler should, in our opinion, be avoided, due to the unknown effects of thermal and mechanical energy deposition in the developing brain [8]. In our experience, using Doppler in the preterm brain rarely alters patient management.

A discussion of normal radiological anatomy is beyond the scope of this article and can be found elsewhere [9]. In our institution, screening of asymptomatic premature neonates born at or before 32 weeks of gestational age consists of CUS examinations at days 1, 7, and 28 of life. The first study, on day 1, aims to identify intraventricular haemorrhage (IVH). The second study, on day 7, assesses for increased echogenicity of the periventricular white matter, suggesting periventricular leukomalacia (PVL). It can also monitor the evolution of IVH identified on the day 1 ultrasound or detect interval haemorrhage. The third study, at day 28, is the most sensitive for cystic PVL and also evaluates for post-haemorrhagic hydrocephalus (PHH) in the setting of prior IVH [2].

## Findings in selected abnormalities

### Preterm brain injury: intraventricular haemorrhage (IVH)

IVH is the most common abnormality identified on neonatal CUS [10].

#### Pathogenesis

The embryological germinal matrix, which lines the ventricular system and produces neuronal and glial cells, reaches its maximum volume at around 25 weeks of gestation and regresses by 36 weeks [11]. The vessels in the germinal matrix, although metabolically active, are immature with thin walls and little structural support [12]. In preterm infants with immature cardiopulmonary systems, hypoxia, hypotension, and fluctuations in cerebral blood flow (CBF) are common and can result in repeated ischaemia-reperfusion episodes [13]. Sudden increases in CBF can rupture fragile germinal matrix vessels, resulting in germinal matrix haemorrhage (GMH), also known as subependymal haemorrhage (SEH). Rupture of the ependymal layer surrounding the germinal matrix (the stria terminalis) can result in extension of haemorrhage into the ventricular lumen, e.g., grade 2 IVH and higher [11]. Research also hypothesises that GMH may be associated with deep medullary vein congestion and thrombosis, which can, in turn, result in white matter necrosis in a pattern similar to PVL [14]. Prematurity is the most significant risk factor for GMH-IVH [15], and there is a proportional relationship between the degree of prematurity and the likelihood of GMH-IVH [16]. GMH-IVH occurs almost exclusively within the first week of life, and is visualised within 72 h of birth in 90% of premature infants [11].

#### Grading and sonographic findings

IVH can be classified as per Papile et al [17], who initially graded IVH on computed tomography (CT), (this has since been modified for use in US), or by Volpe et al [18] (Table 1).

**Table 1:** Grading of IVH

Papile grade [17]	Definition	Volpe grade [18]	Definition
1	Isolated germinal matrix/ subependymal haemorrhage	1	Isolated GMH/SEH
2	Intraventricular haemorrhage without ventricular dilatation	2	IVH without ventricular dilatation (haematoma occupies 10–50% of ventricle)
3	Intraventricular haemorrhage with ventricular distension	3	IVH with acute ventricular dilatation (haematoma occupies > 50% of ventricle)
4	Intraventricular haemorrhage with parenchymal extension	Separate category	IVH with periventricular haemorrhagic infarction

**Fig. 2:**
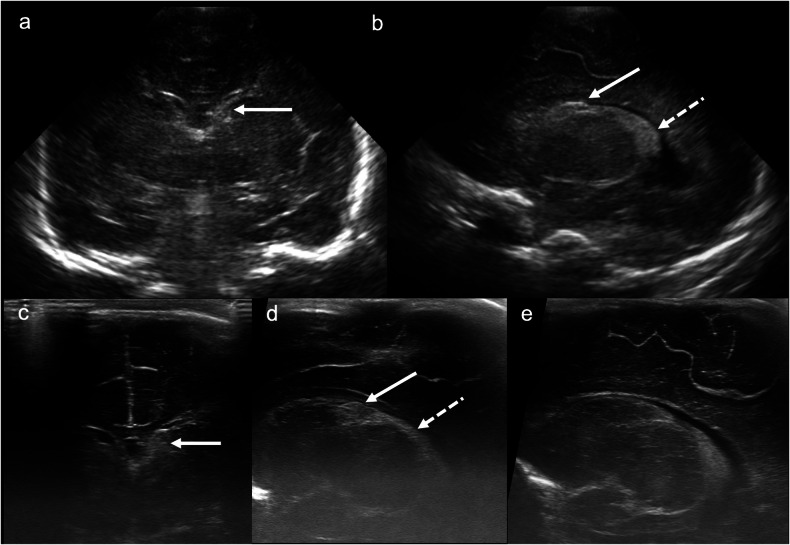
Grade 1 intraventricular/subependymal haemorrhage. **a** Coronal image acquired with a medium-frequency (3–10 mHz) curved probe at the level of the lateral ventricular frontal horns demonstrates echogenic material within the left frontal horn with resultant effacement (solid arrow). **b** Parasagittal image acquired with the same probe as in **a** at the level of the left caudothalamic groove demonstrates echogenic material in the left caudothalamic groove (solid arrow), which is more echogenic than the adjacent choroid plexus (dashed arrow). **c** Coronal image acquired with a high-frequency (10–15 mHz) linear probe at the level of the frontal horns demonstrates effacement of the left frontal horn by echogenic material representing haemorrhage (solid arrow). **d** Parasagittal image acquired with the same probe as in **c** at the level of the left caudothalamic groove demonstrates lack of the normal posterior-to-anterior tapering of the choroid plexus with a “bump” in the caudothalamic groove (solid arrow), which differs slightly in echogenicity from the adjacent choroid plexus (dashed arrow). **e** A normal right caudothalamic groove (parasagittal image) is shown for comparison

Grade 1 IVH refers to an isolated germinal matrix or subependymal haemorrhage. It may be subtle and can easily be overlooked. Parasagittal imaging is often the most helpful in identifying this type of haemorrhage. Diagnostic clues include focal increased echogenicity at the caudothalamic groove, which appears as a “bump” of slightly different echogenicity to the adjacent choroid plexus. The lesion does not taper anteriorly like normal choroid and, when larger, may efface the lateral ventricle. A linear transducer may be helpful in equivocal cases (Fig. 2).

##### Potential pitfalls

**Fig. 3:**
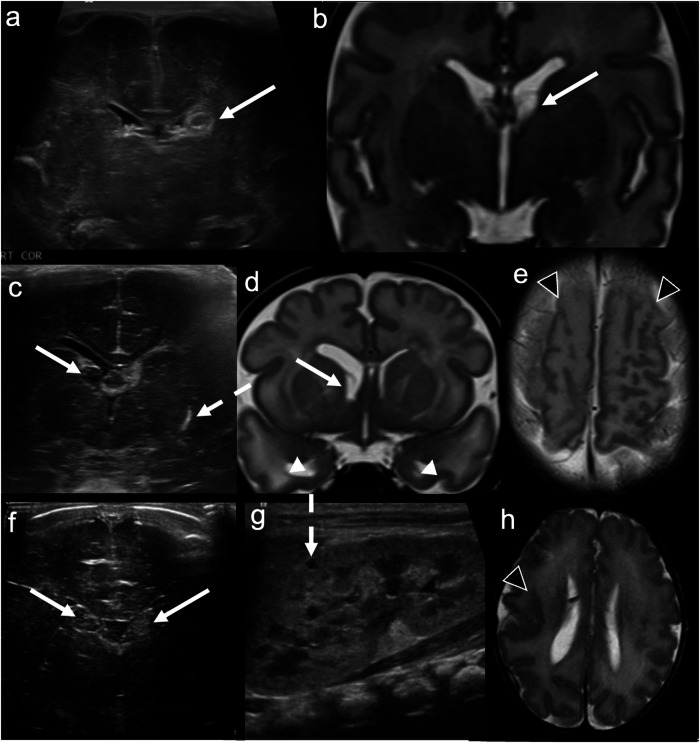
Caudothalamic groove subependymal/germinolytic pseudocysts. Post-haemorrhagic subependymal cyst. **a** Coronal image acquired with a high-frequency (10–15 mHz) linear probe at the level of the frontal horns demonstrates echogenic, expansile material in the left frontal horn, with central hypoechogenicity (solid arrow). Appearances are consistent with grade 1/subependymal haemorrhage with evolving cystic change. **b** Subsequent coronal T2-weighted MRI at the same level demonstrates a simple cyst in the left frontal horn (solid arrow), at the site of the prior subependymal haemorrhage. Congenital cytomegalovirus (CMV) infection. **c** Coronal image acquired with a high-frequency (10–15 mHz) linear probe at the level of the frontal horns demonstrates a cyst in the right frontal horn (solid arrow) in addition to left lenticulostriate mineralising vasculopathy (dashed arrow). **d** Subsequent coronal T2-weighted MRI at the same level demonstrates a simple right frontal horn cyst (solid arrow), as well as bilateral temporal pole cysts (white arrowheads). **e** Axial T2-weighted MRI towards the vertex demonstrates an abnormal, nodular morphology of bilateral frontal lobe gyri, consistent with polymicrogyria (black arrowheads). This constellation of findings—periventricular cysts (especially in the anterior temporal poles), lenticulostriate mineralisation and polymicrogyria—is suggestive of congenital CMV infection, which was proven in this patient. Zellweger syndrome. **f** Coronal image acquired with a high-frequency (10–15 mHz) linear probe at the level of the frontal horns on day 1 of life demonstrates bilateral echogenic, expansile frontal horn cysts (solid arrows), slightly less echogenic than expected for acute subependymal haemorrhage. **g** Sagittal renal ultrasound image in the same patient demonstrates a tiny renal cortical cyst (dashed arrow). **h** Axial T2-weighted MRI at the level of the lateral ventricles demonstrates nodularity of the right peri-Sylvian cortex consistent with polymicrogyria (black arrowhead). This constellation of findings—germinolytic and renal cortical cysts and polymicrogyria—is typical of Zellweger syndrome, which was proven in this patient

Subependymal/germinolytic pseudocysts, most commonly located in the caudothalamic groove, usually result from germinolysis secondary to GMH or microinfarction. Although often incidental, these cysts can also be observed in the context of congenital viral infections (e.g., cytomegalovirus (CMV) and rubella), genetic disorders (e.g., Zellweger or cerebrohepatorenal syndromes (abnormal peroxisomal metabolism)), and metabolic conditions like pyruvate dehydrogenase deficiency. Sonographic findings that raise concern for infective or genetic aetiologies of subependymal/germinolytic cysts include: a cystic appearance on day 1 ultrasound; unexpected evolution of “IVH” on serial CUS; multiple stacked cysts; cysts in a posterior or anterior temporal location; other central nervous system abnormalities (e.g., lenticulostriate vasculopathy); and other systemic abnormalities, such as hepatosplenomegaly or renal cysts (Fig. 3) [19].

**Fig. 4:**
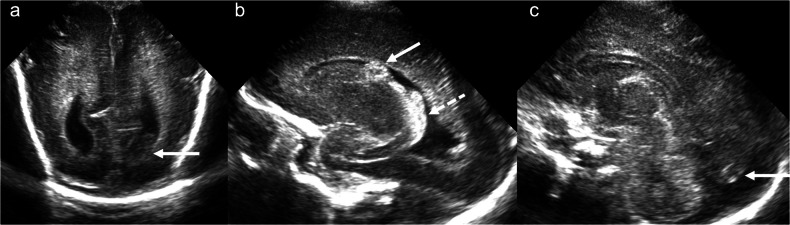
Grade 2 intraventricular haemorrhage. **a** Coronal image acquired with a medium-frequency (3–10 mHz) curved probe at the level of the lateral ventricular occipital horns demonstrates dependent material in the left occipital horn (solid arrow). **b** Parasagittal image acquired with the same probe at the level of the left caudothalamic groove demonstrates bulky echogenic material in the left caudothalamic groove (solid arrow), which is of slightly different echogenicity compared to the adjacent choroid plexus (dashed arrow). **c** Midline sagittal image acquired with the same probe demonstrates echogenic material medially within the occipital horn of the left lateral ventricle (solid arrow)

Grade 2 IVH refers to IVH without ventricular dilatation. In grade 2 IVH, echogenic material, e.g., haematoma, can extend into the frontal horn, anterior to the caudothalamic groove, and/or layer dependently in the occipital horns of the lateral ventricles [20]. This helps to differentiate haematoma from choroid plexus, as the latter does not extend into the lateral ventricular frontal or occipital horns [20]. Occasionally, echogenic choroid plexus can mimic IVH; however, the echogenicity of IVH tends to be slightly different to that of choroid plexus initially and evolves over time. Co-existing layering IVH also helps to differentiate echogenic choroid plexus from IVH intimately related to the choroid. The most medial and posterior aspects of the occipital horns should be carefully scrutinised on every examination to avoid grade 2 IVH being overlooked (Fig. 4). Scanning through the posterior or mastoid fontanelle can improve visualisation of occipital horn IVH [11].

**Fig. 5:**
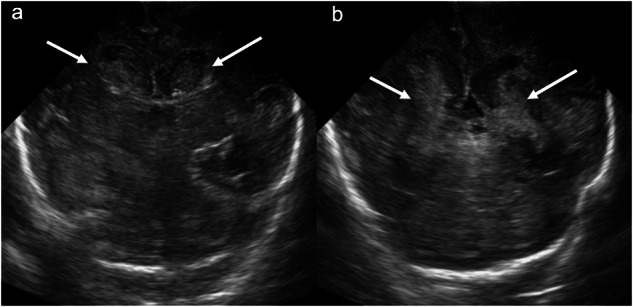
Grade 3 intraventricular haemorrhage. Coronal images acquired with a medium-frequency (3–10 mHz) curved probe at the level of: (**a**) the lateral ventricular frontal horns and (**b**) the lateral ventricular trigones demonstrate bilateral lateral ventricular dilatation secondary to expansile intraventricular haematoma (solid arrows)

Grade 3 IVH is characterised by ventricular dilatation resulting from an expansile clot. This occurs soon after haemorrhage, typically within 1 to 2 days: this contrasts with PHH, which develops after days to weeks as a longer-term sequela (Fig. 5).

**Fig. 6:**
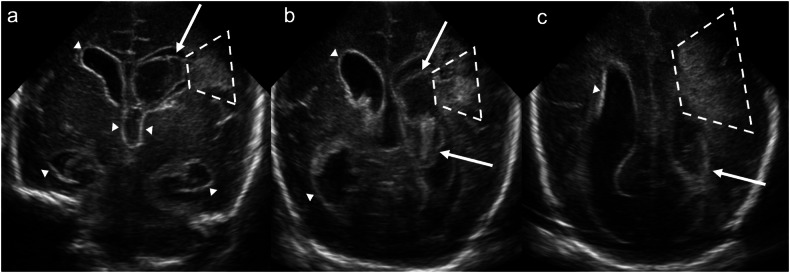
Grade 4 intraventricular haemorrhage/periventricular haemorrhagic infarction. Coronal images acquired with a medium-frequency (3–10 mHz) curved probe at the level of: (**a**) the foramina of Monro, (**b**) the lateral ventricular trigones, and (**c**) the lateral ventricular occipital horns demonstrate a “fan-shaped” area of increased echogenicity in the left frontoparietal periventricular white matter (dashed lines) consistent with periventricular haemorrhagic infarction. Note the expansile haematoma within the left lateral ventricle (solid arrows) and diffuse thickening and increased echogenicity of the ependyma of the lateral and third ventricles (white arrowheads) secondary to prior IVH

Grade 4 IVH was initially described by Papile et al [17] as a direct extension of IVH into the adjacent brain parenchyma. It is now understood that what was once termed “parenchymal haemorrhage” actually reflects periventricular haemorrhagic infarction (PHI) [21] due to venous congestion/obstruction and arteriolar hypoperfusion. Sonographically, grade 4 IVH is characterised by a fan-shaped area of increased echogenicity in the periventricular white matter ipsilateral to the IVH. Initially, the lesion appears separate from the ventricle but later merges with the IVH. Over time, increased echogenicity and oedema resolve, ultimately resulting in cavitation/porencephaly, volume loss and ex vacuo ventricular dilatation (Fig. 6). PHI can occur with any grade of IVH, though it is more common with higher-grade IVH [22]. PHI with an atypical onset, e.g., in the antenatal period or in infants older than 1 week, raises the possibility of a bleeding diathesis or coagulation disorder, e.g., factor V Leiden [22] or other inherited thrombophilia [23]. The pattern of PHI depends on the deep venous outflow pathway involved. For example: congestion/obstruction of the anterior terminal vein results in caudate PHI (anterosuperior to the lateral ventricular frontal horn); congestion/obstruction of the inferior ventricular and lateral atrial vein results in temporal lobe PHI; and congestion/obstruction of a complete terminal vein results in frontoparietal periventricular or deep white matter PHI [24].

The sonographic characteristics of IVH evolve over time. Typically, haematoma is hypo- or iso-echoic in the hyperacute phase (within 4 h of haemorrhage onset), echogenic in the acute phase (4 h to 3 days), and centrally hypoechoic and retractile in the subacute phase (3 days to 4 weeks). After 4 weeks, the haematoma liquefies and further decreases in volume. There may be associated intraventricular debris/septations, and ventricular dilatation (PHH) [22].

**Fig. 7:**
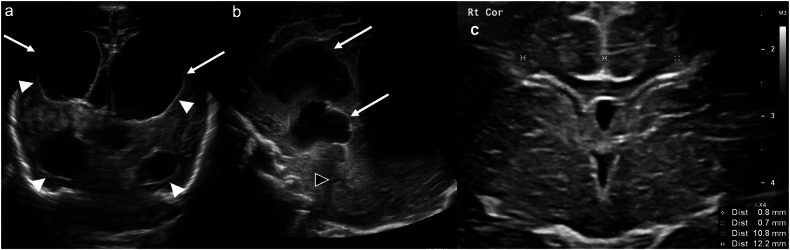
Post-haemorrhagic hydrocephalus. **a** Coronal image acquired with a medium-frequency (3–10 mHz) curved probe at the level of the frontal and temporal horns demonstrates bilateral lateral ventricular dilatation (solid arrows). **b** Midline sagittal image acquired with the same probe as in **a** demonstrates lateral and third ventricular dilatation (solid arrows). The fourth ventricle (black arrowhead) is not dilated (note the partially imaged, asymmetrically dilated left lateral ventricle crossing the midline posteriorly). Findings are in keeping with post-haemorrhagic hydrocephalus, with obstruction at the level of the aqueduct of Sylvius. Again, note the thickened, echogenic ependyma (white arrowheads) secondary to IVH. **c** Coronal image acquired with a high-frequency (10–15 mHz) linear probe at the level of the foramina of Monro illustrates anterior horn width and ventricular index measurements. The anterior horn width refers to the maximal diagonal width of the frontal horn at the level of the foramina of Monro in the coronal plane. The ventricular index refers to the distance from the falx cerebri to the lateral margin of the frontal horn at the same level

PHH typically occurs days to weeks after IVH, most commonly after grade 3 IVH. It can either be communicating (due to impaired cerebrospinal fluid reabsorption) or non-communicating (due to an obstructing clot or fibrin debris). In the case of non-communicating hydrocephalus, the pattern of ventricular dilatation depends on the level of obstruction. Uni- or bilateral obstruction of the foramen/foramina of Monro results in uni- or bilateral lateral ventricular dilatation. Aqueductal obstruction results in bilateral lateral and 3rd ventricular dilatation (Fig. 7). Obstruction of the 4th ventricular outflow tracts (e.g., the foramina of Luschka or Magendie) results in bilateral lateral, 3rd and 4th ventricular dilatation. 4th ventricular entrapment can also occur due to combined aqueductal and 4th ventricular outlet obstruction [22].

Lateral ventricular calibre should be assessed in a standardised manner. The most commonly used measurements are the anterior horn width and the ventricular (Levene) index (Fig. 7c). These metrics have good reproducibility and low interobserver variability [25] and enable early identification and accurate monitoring of evolving PHH of prematurity (PHHoP). This is paramount given that early intervention in PHHoP has been associated with decreased mortality and risk of severe neurodevelopmental disability [26].

**Fig. 8:**
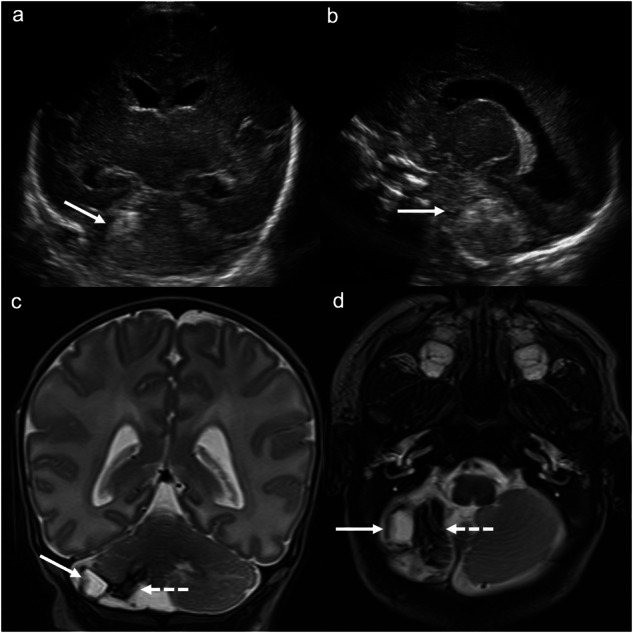
Cerebellar (germinal matrix) haemorrhage. **a** Coronal image acquired with a medium-frequency (3–10 mHz) curved probe at the level of the parahippocampi/medial temporal lobes and (**b**) right parasagittal image acquired with the same probe at the level of the right lateral ventricle demonstrate increased echogenicity in the lateral right cerebellar hemisphere (solid arrows). **c** Coronal and (**d**) axial T2-weighted MRI demonstrate (haemorrhagic) cystic encephalomalacia in the lateral right cerebellar hemisphere (solid arrows) corresponding to the cerebellar haemorrhage on ultrasound. There is low-signal hemosiderin staining medially (dashed arrows)

Cerebellar haemorrhage is an often-overlooked form of GMH. There is a germinal matrix in the external granular layer of the cerebellum and along the roof of the 4th ventricle, which explains the cerebellar hemispheric and/or paravermian haemorrhages that occur in preterm neonates. Cerebellar haemorrhage has recently been categorised by location and type: type 1, punctate haemorrhage without volume loss (most common); type 2, focal haemorrhage with volume loss; type 3, hemispheric ovoid/crescentic haemorrhage, most commonly in the inferior and peripheral cerebellum, and the most commonly missed cerebellar haemorrhage; type 4, vermian haemorrhage; and type 5, large lobar haemorrhage [27]. Transmastoid imaging is often helpful in detecting cerebellar haemorrhage (Fig. 8).

#### Prognosis

There is no definitive prognostic implication with grade 1 or 2 (minor) IVH [28]: the risk of death and neurodevelopmental disability in very preterm infants with these grades of IVH is similar to those without IVH. In contrast, higher-grade IVH (grades 3 and 4) is associated with a proportional increased risk of mortality and neurodevelopmental disability [29].

### Preterm brain injury: periventricular leukomalacia (PVL)

PVL is the second most common form of preterm brain injury.

#### Pathogenesis

PVL is multifactorial but primarily results from a combination of hypoxia-ischaemia and inflammation. The periventricular white matter of the preterm infant is particularly susceptible to ischaemia due to its immature vascularisation and arterial border zone location; this is compounded by immature CBF autoregulation and an inability to compensate for fluctuations in blood pressure. Immature oligodendrocytes in the white matter of preterm infants, which are crucial for normal brain development (including myelination), are susceptible to hypoxia-ischaemia and inflammation, and insult to these cells can result in failure of maturation or death. In addition, inflammation causes neurotoxic cytokines to be released and is associated with dysfunctional activation of microglia, which can adversely affect cortical development [30].

#### Grading and sonographic findings

PVL was classified on CUS in 1992 by de Vries et al [31] (Table 2).

**Table 2:** Grading of PVL [31]

Grade	Definition
1	Periventricular echogenicity persisting for ≥ 7 days
2	Periventricular echogenicity evolving into localised frontoparietal cysts
3	Extensive periventricular cystic lesions (cystic PVL)
4	Extensive cystic lesions in the deep white matter

One of the early sonographic clues to PVL is heterogeneity and increased echogenicity of the periventricular white matter. The degree of heterogeneity is more important than the degree of increased echogenicity. Importantly, the widely held belief that periventricular white matter must appear more echogenic than adjacent choroid plexus is inaccurate, given that the highest echogenicity on US does not correlate with, nor predict, the severity of white matter injury on MRI [32].

**Fig. 9:**
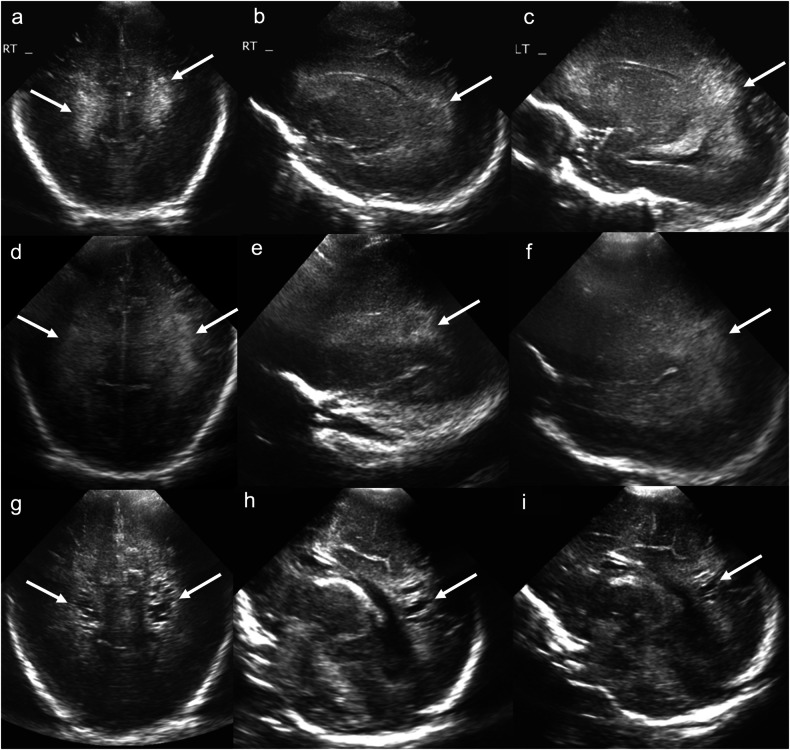
Periventricular leukomalacia. Coronal images acquired with a medium-frequency (3–10 mHz) curved probe at the level of the parietal and occipital lobes (**a**, **d**, **g**) and right (**b**, **e**, **h**) and left (**c**, **f**, **i**) parasagittal images acquired with the same probe at the level of the lateral ventricles on days 1 (**a**–**c**), 14 (**d**–**f**) and 28 (**g**–**i**) of life in a 31-week preterm neonate. **a**–**c** Note heterogenous increased echogenicity of the posterior periventricular white matter bilaterally (solid arrows). **d**–**f** This heterogenous white matter increased echogenicity (left greater than right) persisted on the follow-up ultrasound 2 weeks later. **g**–**i** At day 28, there has been interval development of bilateral occipital and posterior periventricular cysts with persistent increased echogenicity of the surrounding white matter consistent with cystic PVL

Cystic PVL is usually evident by 3 weeks of age. However, in up to 18% of neonates, the cystic lesions of PVL disappear by term-equivalent age [33]. Cystic PVL, as visualised on CUS, is characterised by simple/anechoic periventricular white matter cysts that are separate from the ventricles, with surrounding increased echogenicity. The ependymal contour of the adjacent lateral ventricles may be irregular due to white matter volume loss (Fig. 9) [34].

##### Potential pitfalls

**Fig. 10:**
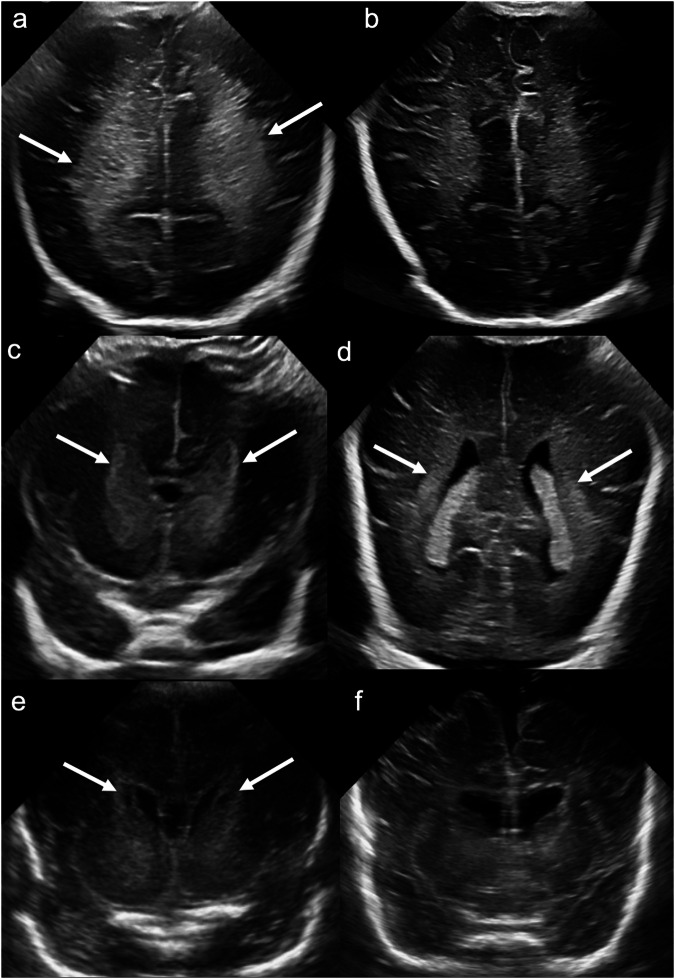
Periventricular flare. **a** Coronal image acquired with a medium-frequency (3–10 mHz) curved probe at the level of the parietal and occipital lobes on day 1 of life in a preterm infant demonstrates bilateral, symmetrical, homogenous increased echogenicity of the posterior white matter (solid arrows). **b** Coronal image at the same level on day 28 ultrasound demonstrates normalisation of parieto-occipital white matter echogenicity. Term-equivalent MRI (not shown) demonstrated normal periventricular white matter signal, without cystic change. Normal white matter tracts. **c**, **d** Coronal images acquired with a medium-frequency (3–10 mHz) curved probe at the level of: (**c**) the lateral ventricular frontal horns and (**d**) the lateral ventricular trigones demonstrate bilateral, symmetrical, increased echogenicity along the anterior limbs of internal capsules (**c**) and optic radiations (**d**) respectively (solid arrows), consistent with normal white matter tracts. Connatal cysts. **e** Coronal image acquired with a medium-frequency (3–10 mHz) curved probe at the level of the frontal horns demonstrates bilateral periventricular cysts situated anterior to the foramina of Monro and below the superior margin of the frontal horns (solid arrows). **f** Coronal image at the same level on an ultrasound performed at 4 months corrected age (for increasing head circumference) demonstrates resolution of the periventricular cysts

Transient periventricular hyperechogenicity, also referred to as periventricular “flare”, is thought to occur due to venous congestion [35], is common in preterm infants and more pronounced with lower gestational age [36]. Unlike PVL, periventricular “flare” appears homogenously echogenic and typically resolves within 1 week (Fig. 10a, b) [35].

Bilateral, symmetrical increased echogenicity within the frontal and peritrigonal white matter reflects the white matter tracts of the anterior limbs of internal capsules and optic radiations, respectively: it is a normal finding and should not be mistaken for PVL (Fig. 10c, d) [34].

Frontal horn cystic lesions are typically incidental and of doubtful clinical significance. Connatal cysts are ependymal-lined cysts of the inner ventricular wall, whereas subependymal/germinolytic pseudocysts at the caudothalamic groove arise from the frontal horn germinal matrix [37]. Connatal cysts are typically present at birth, and visible on day 1 ultrasound. They are ovoid/ellipsoid or triangular in shape, and are located at the junction between the frontal horn and the lateral ventricular body, anterior to the foramina of Monro and inferior to the superior margin of the frontal horn. They usually abut and “fit into” the lateral ventricular margin and contact the ependyma without intervening tissue. They typically resolve by 1 to 2 months of age (Fig. 10e, f) [37, 38]. Frontal horn cystic lesions should be distinguished from frontal white matter necrosis, which tends to be larger, more irregular, and extends beyond the frontal horn margins, often superiorly. When chronic, it is associated with volume loss, ex vacuo ventricular dilatation, and gliosis [37].

Periventricular cysts can be seen in the setting of CMV infection, especially in the anterior temporal regions. When adjacent to the trigones or occipital horns of the lateral ventricles, these can mimic cystic PVL (Fig. 3c–e).

#### Prognosis

Given that cystic PVL most commonly affects the posterior/peritrigonal white matter, where the lower limb motor, somatosensory and posterior visual pathways are located, the long-term sequelae of PVL include visual, auditory and motor deficits with spastic diplegia or quadriplegia. The presence and severity of cystic change are the most reliable predictors of poor neurologic outcomes, i.e., cerebral palsy [34]. Infants with apparently “resolved” cystic PVL remain at increased risk of neurodevelopmental impairment [33].

A checklist for reporting the preterm brain is included in Table 3.

**Table 3:** Reporting checklist for the preterm brain

Structure	Sub-region	Findings	Mimics
**Lateral ventricles**	**Caudothalamic groove**	**Subependymal haemorrhage:** • “Bump” • Echogenicity differs from choroid plexus • Does not taper smoothly like normal choroid plexus	**Caudothalamic groove subependymal/germinolytic cysts**: • Anechoic/hypoechoic at birth (unlike hyperechoic acute haemorrhage)
	**Occipital horns**	**Intraventricular haemorrhage:** • Echogenic layering of haemorrhage • Occipital horns curve medially; this portion must be assessed	
	**Frontal horns**	**Ventricular dilatation:** • Subjective: Early lateral convexity of the frontal horns • Objective: Increased anterior horn width/ventricular index	
	**Trigones**	**Ventricular irregularity:** • Suggests white matter volume loss secondary to periventricular leukomalacia (PVL)	
**White matter**	**Periventricular white matter**	**Echogenicity**	
		**Early PVL:** • Increased echogenicity of peritrigonal white matter • Heterogeneity more important than degree of echogenicity	**Periventricular flare**: • Transient, resolves by day 7 • Homogeneously echogenic **Normal optic radiations:** • Symmetrical, parasagittal echogenicity, linear when traced anteriorly **Normal internal capsules:** • Symmetrical, linear parasagittal echogenicity
		**Periventricular haemorrhagic infarction (PHI):** • Not extension of IVH into parenchyma • Can occur with any grade IVH • Fan-shaped echogenicity, ipsilateral to IVH, slightly separate from lateral ventricle initially • Positive mass effect, unlike PVL • Echogenicity correlates with deep vein affected	
		**Cystic change**	
		**Cystic PVL:** • Evident by 3 weeks of age • Anechoic periventricular cysts, separate from ventricles, surrounded by halo of increased echogenicity	**Connatal cysts:** • Incidental, evident from day 1 • Ovoid/ellipsoid or triangular, anterior to foramen/foramina of Monro, limited to upper margin of frontal horn ependyma **Periventricular pseudocysts in CMV:** • Evident from day 1 • Multiple, stacked cysts, anterior temporal a typical location
		**Cavitating PHI** • Central cystic change within PHI • Cavitation reflects cystic encephalomalacia and volume loss late in haemorrhage evolution	
**Cerebellum**	**Hemispheres, paravermian**	**Cerebellar haemorrhage:** • A form of GMH • Focal, asymmetric echogenicity, can be large with positive mass effect	

### Future directions

Emerging techniques, such as ultrafast US, may add value to conventional imaging methods. The development of ultrafast US Doppler has enabled quantification of cerebrovascular resistivity, which has been shown in preterm neonates to predict white matter lesion burden at term-equivalent MRI [39]. Additionally, shear wave elastography has been used to quantitatively measure brain stiffness in preterm neonates and has demonstrated increasing tissue elasticity with gestational age, lower tissue elasticity in preterm neonates with evidence of white matter injury on conventional US, and lower tissue elasticity on day 3 of life US in preterm neonates with evidence of white matter injury on subsequent term-equivalent MRI [40]. While these techniques require further validation, they hold promise as early markers of white matter injury in preterm neonates. However, the potential risks associated with these new sonographic techniques, with regard to the thermal and mechanical effects on the developing brain, must be taken into consideration.

Although not yet clinically applicable, artificial intelligence could enhance the use of CUS in the preterm brain. Potential applications include early detection of ventricular dilatation in the context of PHHoP, which can be difficult to diagnose without quantification, as well as screening for structural brain abnormalities [41].

## Conclusion

Cranial US is an invaluable tool in assessing the neonatal brain. It can be used in preterm infants to diagnose, monitor and assess for complications arising from IVH and PVL. However, caution should be exercised when obtaining and interpreting sonographic images to avoid common pitfalls that may mimic IVH and PVL, and which can have implications for management, prognostication and discussions with parents/caregivers.

## Data Availability

The data will not be made publicly available due to privacy restrictions.

## Abbreviations

CBF: Cerebral blood flow
CMV: Cytomegalovirus
CUS: Cranial US
GMH: Germinal matrix haemorrhage
IVH: Intraventricular haemorrhage
PHH: Post-haemorrhagic hydrocephalus
PHHoP: Post-haemorrhagic hydrocephalus of prematurity
PHI: Periventricular haemorrhagic infarction
PVL: Periventricular leukomalacia

## Declarations

### Guarantor

Dr Rahul Lakshmanan, Medical Imaging Department, Perth Children’s Hospital, 15 Hospital Avenue, Nedlands, WA 6009, Australia. Telephone: +61 8 6456 2222. Facsimile: +61 6 6456 0072. E-mail: rahul.lakshmanan@health.wa.gov.au.
